# Alcoholic Beverages as a Source of Estrogens

**Published:** 1998

**Authors:** Judith S. Gavaler

**Affiliations:** Judith S. Gavaler, Ph.D., is a professor at the Oklahoma Medical Research Foundation and an adjunct professor of biostatistics and epidemiology at the University of Oklahoma College of Public Health, Oklahoma City, Oklahoma

**Keywords:** alcoholic beverage, congener, estrogens, male, female, plant, alcoholic liver cirrhosis, testicular dysfunction, feminization, hypothesis testing, biochemical mechanism, animal model, ovary, moderate AOD use, prolactin, follicle stimulating hormone, luteinizing hormone, cholesterol, globulins, literature review

## Abstract

Alcoholic beverages contain not only alcohol but also numerous other substances (i.e., congeners) that may contribute to the beverages’ physiological effects. Plants used to produce alcoholic beverages contain estrogenlike substances (i.e., phytoestrogens). Observations that men with alcoholic cirrhosis often show testicular failure and symptoms of feminization have suggested that alcoholic beverages may contain biologically active phytoestrogens as congeners. Biochemical analyses have identified several phytoestrogens in the congeners of bourbon, beer, and wine. Studies using subjects who produced no estrogen themselves (i.e., rats whose ovaries had been removed and postmenopausal women) demonstrated that phytoestrogens in alcoholic beverage congeners exerted estrogenlike effects in both animals and humans. Those effects were observed even at moderate drinking levels.

The excessive consumption of alcoholic beverages is associated with numerous serious medical, social, and legal problems that exact a high human and economic price. Despite the monumental problems caused by alcohol abuse and dependence, however, the fact is that most people who drink consume moderate amounts of alcoholic beverages. Indeed, an additional direction for alcohol research has been generated by reports of the beneficial effect of moderate drinking on the risk of coronary heart disease (CHD) (Klatsky 1994). Thus, studies have found that compared with abstainers and heavier drinkers, moderate drinkers (i.e., women who consume up to one standard drink^1^ per day and men who consume up to two drinks per day) have a significantly reduced risk of CHD. This definition of moderate drinking also includes people who consume alcoholic beverages only occasionally and corresponds to the recommended limits for low-risk alcohol consumption (U.S. Department of Agriculture and U.S. Department of Health and Human Services 1995).

When discussing the risks and benefits associated with alcoholic beverages, most people think in terms of the beverages’ alcohol contents. Consequently, much of the research aimed at determining how alcoholic beverages affect the body has been conducted using alcohol solutions to approximate the effects of alcoholic beverages. Alcoholic beverages, however, contain numerous substances in addition to alcohol itself (i.e., congeners), which determine a beverage’s taste, color, and aroma. Alcoholic beverages differ in both the composition and quantity of congeners. These variations result from the different methods and materials (e.g., grains, fruits, and hops) from which the beverages are produced. This article explores the hypothesis that congeners, particularly phytoestrogens, contribute to the effects of alcoholic beverages on the body.

## Evidence of the Estrogenic Activity of Congeners

Researchers’ interest in the congeners of alcoholic beverages first was spurred by various reports in the agricultural literature. For example, some studies reported that grazing animals feeding on particular forages and grasses showed evidence of impaired reproduction. Subsequently, using those forages, researchers isolated substances that exhibited estrogenlike activity and later identified them as estrogenlike substances of plant origin (i.e., nonsteroidal phytoestrogens) (see the following section). Finally, studies demonstrated that the phytoestrogens found in milled by-products and oils made from various grains, hops, corn, and rice exhibited biological activity both in experimental animals and in studies using cultured cells (see Gavaler et al. 1987*a*,*b*; 1995*a*,*b*; and references therein).

### Estrogens and Their Activities

Estrogens are female sex hormones that are produced primarily in the ovaries. These hormones play essential roles in the development and maintenance of the female reproductive organs and breasts as well as in pregnancy and lactation. In men, small amounts of estrogens are produced in the testes. (For more information on estrogens and their functions, see the article by Hiller-Sturmhöfel and Bartke, pp. 153–164.) Based on the structure of the molecules, two main classes of estrogens exist: steroidal and nonsteroidal. The steroidal estrogens—estradiol and estrone—are generated by the body. Nonsteroidal estrogens, also known as phytoestrogens, are produced by certain plants.

Estrogens exert their effects by entering their target cells, where they bind to docking molecules (i.e., receptors) in the fluid that fills the cell (i.e., the cytosol). The estrogen-receptor complexes then move into the cell nucleus, where the transported estrogen is transferred to nuclear estrogen receptors. The nuclear complexes, in turn, interact with a certain type of genetic molecule (i.e., ribonucleic acid [RNA]) in the nucleus, thereby influencing the activity of certain genes and modifying the cell’s function. The strength (i.e., specificity and affinity) with which estrogens bind to their receptors differs among the molecules. Thus, steroidal estrogens exhibit greater affinity and specificity for estrogen receptors than do nonsteroidal phytoestrogens (Rosenblum et al. 1993; Hertog et al. 1993; Miksicek 1995; Makela et al. 1995) and therefore generally are more powerful in their actions.

### Phytoestrogens in Alcoholic Beverages—A Hypothesis

Case studies have shown that men with liver damage resulting from excessive alcohol consumption (i.e., alcoholic cirrhosis) often suffer from testicular failure—the inability of the testes to produce male sex hormones. In addition, those men also frequently show signs and symptoms of feminization, such as enlarged breasts and a redistribution of body fat into a pattern that mimics that of women (for reviews, see Wright et al. 1992; Gavaler and Van Thiel 1988). These signs and symptoms are consistent with exposure to high levels of estrogen. Surprisingly, however, the levels of the steroidal estrogens in cirrhotic, feminized men are similar to or only slightly elevated compared with the levels in age-matched nonalcoholic men.

The fact that alcoholic beverages are made from many plants and plant byproducts that contain phytoestrogens has led to the hypothesis that alcoholic beverages contain biologically active phytoestrogens as congeners. According to this hypothesis, two factors might contribute, at least in part, to the feminization observed in men with alcoholic cirrhosis: (1) prolonged exposure to the phytoestrogens contained in alcoholic beverage congeners and (2) the impaired ability of the alcohol-damaged liver to adequately metabolize and excrete many compounds, including phytoestrogens. The hypothesis has been tested in biochemical analyses, animal models, and human studies. The results of those analyses are summarized in the following sections.

## Biochemical Analyses

**Figure 1:**
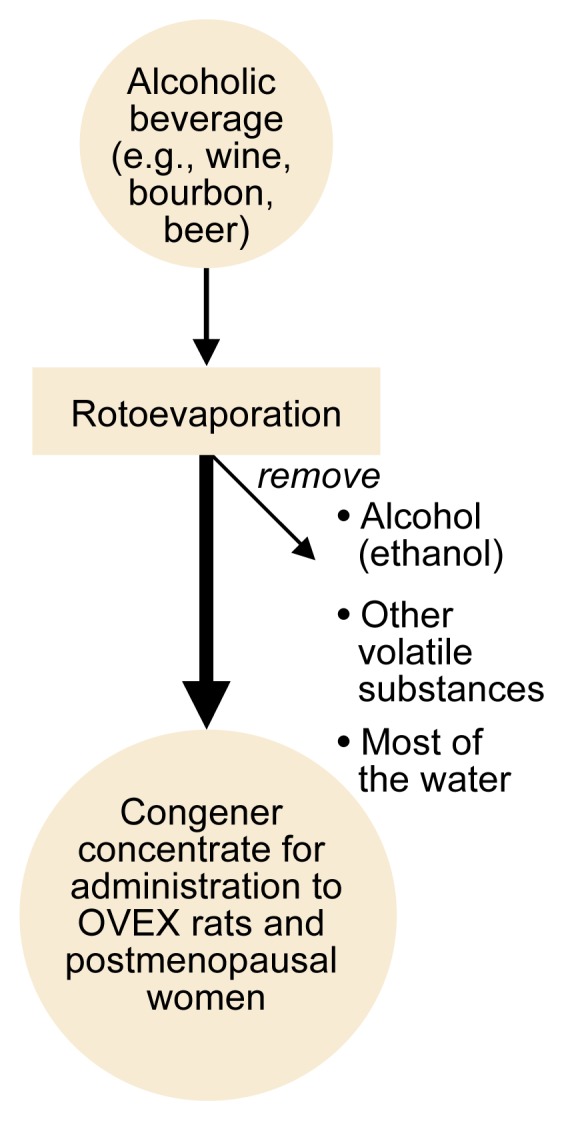
Schematic representation of the preparation of alcoholic beverage congener concentrates, which can be used to investigate the effects of those congeners in rats whose ovaries have been removed (OVEX rats) and in postmenopausal women.

To investigate the hypothesis that some of the effects of alcoholic beverages result from estrogenic congeners, researchers removed all of the alcohol, other volatile substances, and most of the water contained in various alcoholic beverages (e.g., bourbon, wine, and beer) using a technique called rotoevaporation (see figure 1). The resulting congener concentrates then were subjected to sophisticated biochemical analyses, such as gas chromatography and mass spectrometry, to isolate and identify any estrogenic compounds present. Those analyses identified two phytoestrogens—sitosterol and biochanin A—in bourbon; two additional phytoestrogens—daidzein and genistein—were present in beer (Rosenblum et al. 1987, 1991). Other phytoestrogens have been identified in wine (Hertog et al. 1993).

In addition, both the congener concentrates and the purified phytoestrogens were examined for their ability to bind to estrogen receptors and to compete with estradiol for binding to estrogen receptors in the cytosol (Gavaler et al. 1987*b*; Hertog et al. 1993; Makela et al. 1995). Those analyses found that compared with estradiol, phytoestrogens bound less strongly to the estrogen receptors and were less able to compete for binding to the receptors. It is important to note, however, that although those studies were able to determine the relative ability of phytoestrogens to interact with estrogen receptors in the cytosol, they provided no information about whether the molecules also were transported to the nucleus and bound to nuclear receptors.

## Experimental Analyses of the Role of Phytoestrogens in Animal Models

### The Experimental Animal Model

To maximize the probability of detecting a response to biologically active phytoestrogens in alcoholic beverage congeners, researchers needed to use animals that produced no or very little estrogen themselves. One such model is a female rat whose ovaries have been removed (i.e., an OVEX rat). In the absence of the ovaries and thus of cyclic ovarian function, the animal’s estrogen production is greatly diminished. (This type of rat also can serve as a model for postmenopausal women, whose ovaries have ceased to produce estrogen.)

The loss of ovarian hormone production induces several changes in the body that can serve as markers of the level of ovarian function. First, the body substantially increases the levels of certain hormones (i.e., gonadotropins) that regulate ovarian estrogen production and secretion, thereby attempting to enhance estrogen production by the unresponsive, or absent, ovaries. Two gonadotropins exist—follicle-stimulating hormone (FSH) and luteinizing hormone (LH)—whose levels in the blood can be measured easily. Thus, when OVEX rats are exposed to exogenously administered estrogenic substances, such as phytoestrogens, the levels of both FSH and LH would be expected to decline compared with unexposed control animals, because the body would no longer need to stimulate ovarian activity to the same extent. Second, estrogen deprivation results in shrinkage, or atrophy, of the uterus and fallopian tubes, the extent of which can be measured by determining those organs’ weights. Accordingly, exposure to phytoestrogens would be expected to increase the weight of the uterus (which in the rat includes the fallopian tubes) compared with untreated animals.

**Figure 2:**
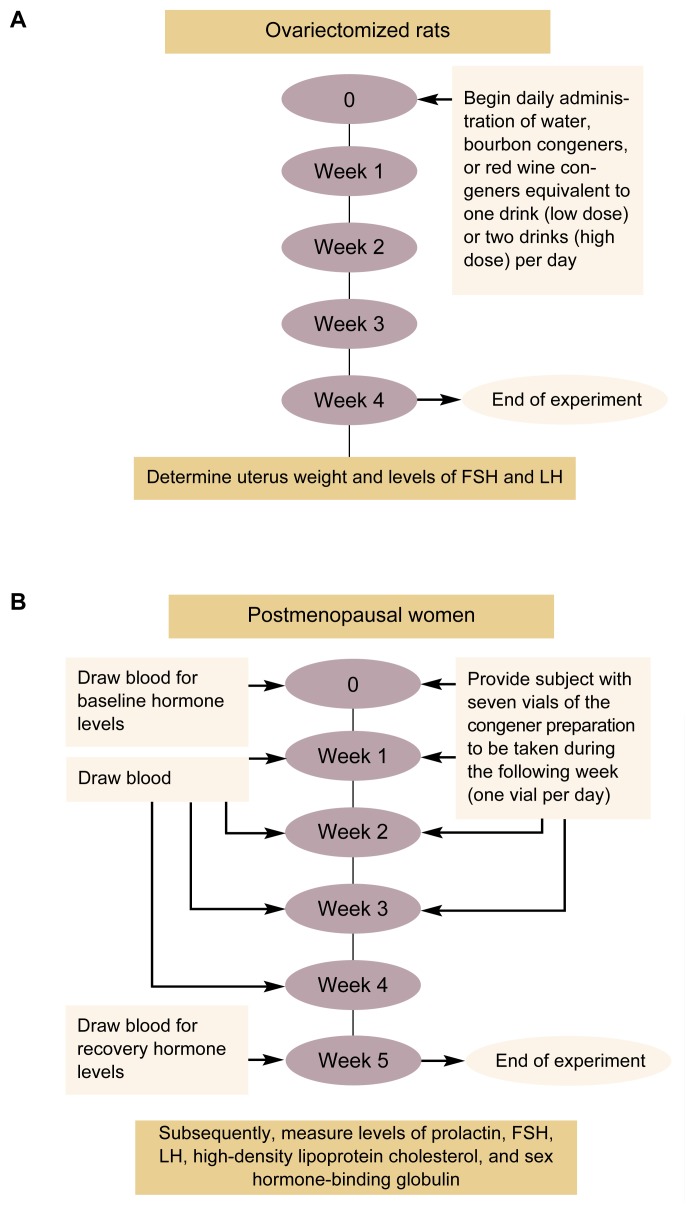
Protocol of experiments to evaluate potential estrogenlike effects of alcoholic beverage congeners in (A) rats whose ovaries have been removed (i.e., ovariectomized) and (B) postmenopausal women. NOTE: FSH = follicle-stimulating hormone; LH = luteinizing hormone.

For the experiments with OVEX rats, concentrates of red wine and bourbon congeners were prepared the same way as for the biochemical analyses described in the previous section. The concentrates then were diluted so that 100 milliliters (mL) of the animals’ drinking water contained the amount of congeners present in one (low-dose) or two (high-dose) standard drinks of each type of alcoholic beverage. The OVEX rats received the congener-supplemented drinking water daily for 4 weeks (see figure 2). Control animals received plain drinking water. After the experimental period, the animals’ uterus weight and LH and FSH levels were determined.

### Results

**Figure 3:**
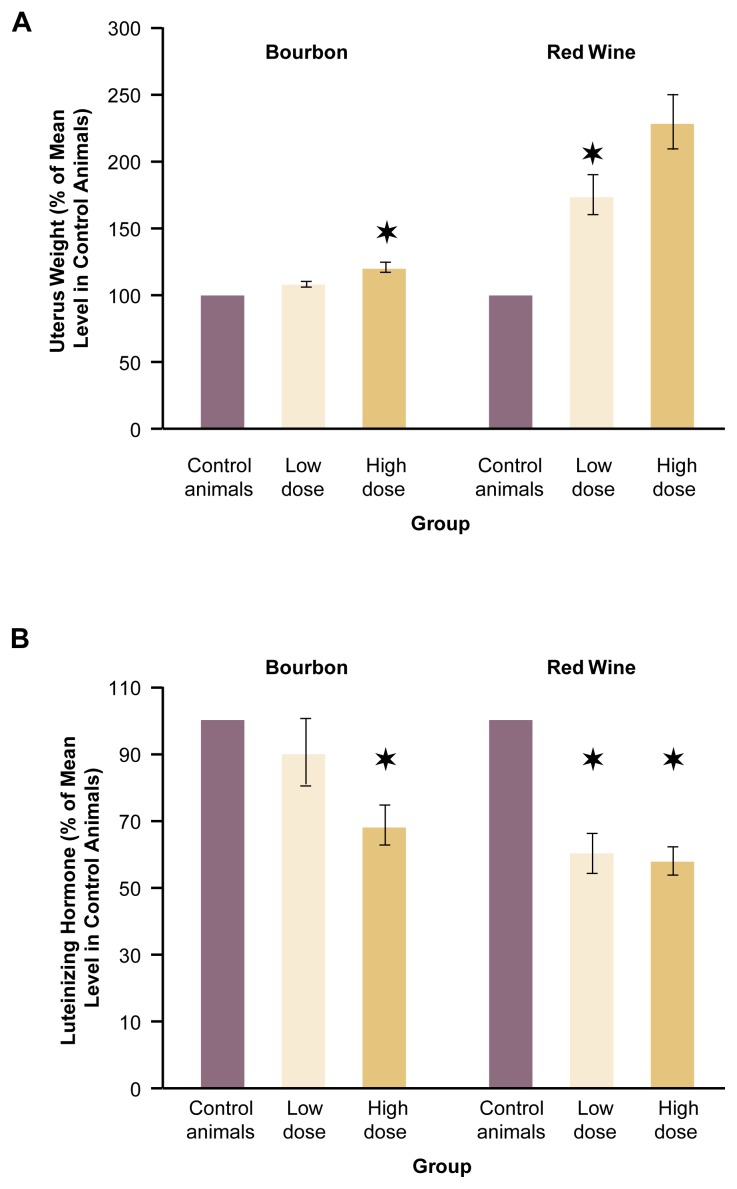
The effects of bourbon and red wine congeners on (A) uterus weight and (B) luteinizing hormone (LH) levels of rats whose ovaries had been removed. The animals received congeners corresponding to one standard drink (low dose) or two standard drinks (high dose) daily in their drinking water for 4 weeks. Uterus weights and LH levels in the congener-exposed animals are expressed as the percentage of the mean level in unexposed control animals (defined as 100 percent). The uterus weights are corrected for the animals’ body weights. Both bourbon and red wine congeners induced estrogenlike effects (i.e., increased uterus weight and reduced LH levels). Moreover, red wine congeners induced more pronounced changes than did bourbon congeners. NOTE: The wide bars represent mean values, whereas narrow brackets represent the standard error of the mean. A star above a bar indicates a statistically significant difference from the value in the control animals (*p* < 0.05).

The congeners of both bourbon and red wine exerted a dose-dependent estrogenic effect on the OVEX rats’ uterus weights and LH levels (see figure 3) (Gavaler et al. 1987*b*, 1995*a*). Thus, the animals’ mean uterus weights increased, and the LH levels decreased compared with control OVEX rats that had received no congeners. Interestingly, the estrogenic effects on both uterus weight and LH levels were more pronounced in the animals exposed to red wine congeners than in the animals exposed to bourbon congeners. In addition, the estrogenic effects reached statistical significance at lower doses of red wine congeners than of bourbon congeners. These findings suggest that red wine contains a higher content and/or biologically more active phytoestrogens than does bourbon.

## Experimental Analyses of the Role of Phytoestrogens in Humans

As described in the previous section, the congeners of two types of alcoholic beverages, bourbon and red wine, produced detectable estrogenic effects in OVEX rats, thereby confirming the hypothesis that alcoholic beverages contain biologically active estrogenic substances. These findings also support the notion that the feminization observed among men with alcoholic cirrhosis might result, at least in part, from high exposure to biologically active estrogenic substances in alcoholic beverages. Because most people consume only moderate amounts of alcoholic beverages, however, it is reasonable to ask whether biologically active estrogenic congeners could elicit a clinically relevant response in humans even at moderate drinking levels.

Three sets of reports, when evaluated together, support the idea that alcoholic beverages may also produce estrogenic effects in moderate drinkers. First, numerous studies have demonstrated a reduced risk of CHD in postmenopausal women who use estrogen replacement therapy. One postulated mechanism underlying this effect involves the ability of estrogen to increase levels of high-density lipoprotein (HDL) cholesterol, or “good” cholesterol. Second, as described previously, several studies have reported a decreased risk of CHD among moderate drinkers (Klatsky 1994). Finally, according to the so-called French Paradox, French people have a low mortality rate from CHD despite a high intake in saturated fats. This effect has been attributed to the French people’s relatively high consumption of alcoholic beverages in general and of red wine in particular (Bordeaux Symposium 1998). When analyzed together, these studies suggest that moderate alcohol consumption can have effects similar to estrogen replacement therapy in terms of reducing the drinker’s risk of CHD. To investigate this issue in more detail, researchers have used a human model equivalent to the OVEX rats (i.e., post-menopausal woman) to evaluate the hypothesis that alcoholic beverage congeners contain biologically active estrogenic substances that produce a clinically significant effect even at moderate drinking levels.

### The Human Study Sample

The study recruited 24 postmenopausal women ranging from ages 57 to 59 (46 percent from minority populations) who were not using estrogen replacement therapy (i.e., were estrogen deficient). The women were all light social drinkers or abstainers and consumed no alcohol during the study. The study evaluated the effects of four types of alcoholic beverage congeners (i.e., from white and red wine, beer, and bourbon) in the women. Thus, six women received white wine congeners, seven women received red wine congeners, six women received beer congeners, and five women received bourbon congeners. Every evening for 4 weeks, each woman consumed a congener preparation corresponding to one standard drink of that beverage. To evaluate whether the women showed a clinically relevant response indicating exposure to biologically active estrogenic substances in the congener preparations, the researchers measured the levels of several hormones and other proteins. These included three hormones produced in the pituitary gland (i.e., FSH, LH, and prolactin^2^) and two estrogen-responsive proteins produced by the liver (i.e., HDL cholesterol and sex hormone-binding globulin [SHBG]). If the congeners contained biologically active phytoestrogens, the FSH and LH levels should decrease as described previously, whereas prolactin levels should increase. Similarly, the levels of HDL cholesterol and SHBG should increase following exposure to estrogenic alcoholic beverage congeners.

To determine the baseline levels of the markers before the women were exposed to the congener preparations, blood samples were obtained at the onset of the study (see figure 2, p. 222). During the 4-week period of daily congener ingestion, blood samples were drawn each week. In addition, the subjects were weighed weekly. Finally, blood samples were obtained 1 week after the women had stopped ingesting the beverage congeners to determine whether the amounts of hormones and binding proteins had returned to baseline levels. Thus, each woman served as her own control. The researchers then could determine the highest (i.e., peak) or lowest (i.e., trough) levels of proteins and hormones achieved during the 4-week study period as well as the levels after exposure had been terminated for 1 full week (i.e., the recovery level). For each study participant, the values then were compared with the corresponding levels before congener exposure began.

### Results

**Figure 4:**
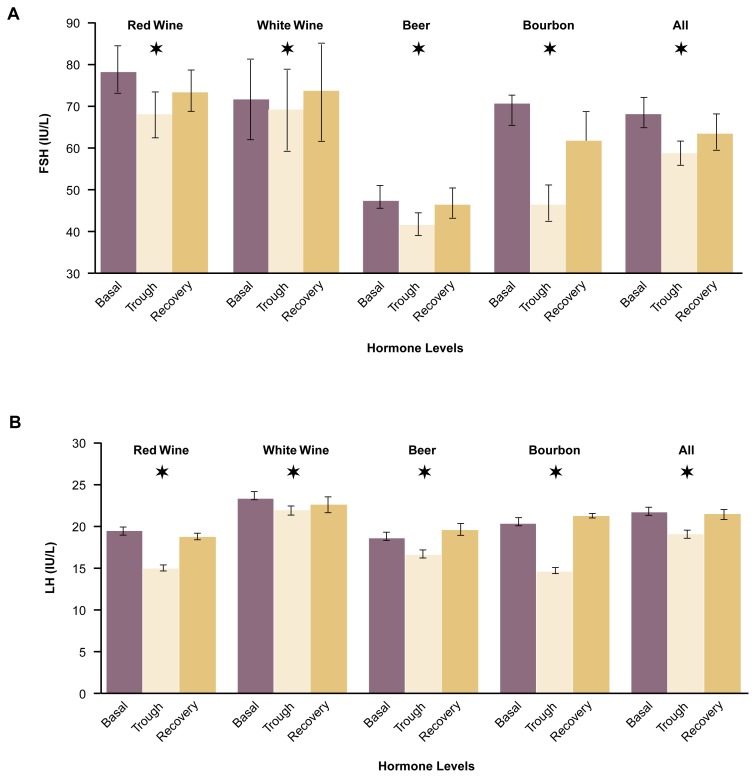
Effects of alcoholic beverage congeners on (A) follicle-stimulating hormone (FSH) and (B) luteinizing hormone (LH) levels in postmenopausal women. For 4 weeks, the women consumed congener amounts corresponding to those present in one standard drink of the beverage daily. Basal hormone levels were determined before the women began the experiment. Trough levels represent the lowest hormone levels that were detected during the 4-week administration period of alcoholic beverage congeners. Recovery levels were determined 1 week after the last ingestion of congeners. All congeners had estrogenlike effects (i.e., resulted in lower FSH and LH levels). The effects of the various congeners did not differ significantly. NOTE: The wide bars represent mean values, whereas the narrow brackets represent the standard error of the mean. A star above a bar indicates a significant difference from basal levels as determined by paired T-test (*p* < 0.025). The differences in baseline levels result from variations in the mean levels of the subjects in the various groups. IU/L = International units per liter.

**Figure 5:**
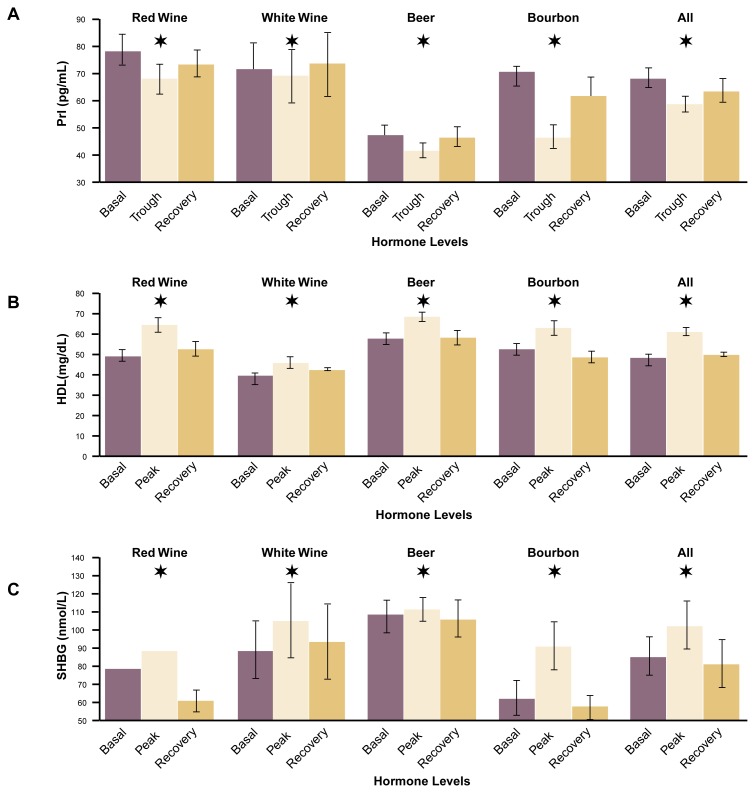
Effects of alcoholic beverage congeners on the levels of (A) prolactin (Prl), (B) high-density lipoprotein (HDL) cholesterol, and (C) sex hormone-binding globulin (SHBG) in postmenopausal women. For 4 weeks, the women consumed congener amounts corresponding to those present in one standard drink of the beverage daily. Basal hormone levels were determined before the women began the experiment. Peak levels represent the highest hormone levels that were detected during the 4-week administration period of alcoholic beverage congeners. Recovery levels were determined 1 week after the last ingestion of congeners. All congeners had estrogenlike effects (i.e., resulted in elevated levels of Prl, HDL cholesterol, and SHBG). The effects of the various congeners did not differ significantly.

For all the hormones and other proteins examined, the levels changed as would be expected if the congeners contained biologically active phytoestrogens (see figures 4 and 5, p. 225) (Gavaler et al. 1995*a*). Thus, the levels of FSH and LH decreased, with trough levels significantly lower than baseline levels. Conversely, the levels of prolactin, HDL cholesterol, and SHBG increased during the study period and reached peak levels that were significantly higher than the baseline levels. The women’s weights did not change over the study period. In addition, following the recovery period of 1 week, the levels of all five markers returned to values that did not differ significantly from baseline levels. No statistically significant differences existed in the estrogenic effects of the various congener concentrates.

## Conclusions and Future Directions

The studies described in the previous sections strongly support the hypothesis that congeners present in alcoholic beverages can produce measurable estrogenic effects, even at moderate drinking levels. Specifically, those studies found the following:

- Alcoholic beverage congeners exerted estrogenic effects both in an experimental animal model and in post-menopausal women.
- The estrogenic effects of alcoholic beverage congeners were detectable using a variety of estrogenic markers, including the pituitary hormones LH (in OVEX rats and postmenopausal women), FSH, and prolactin (in postmenopausal women); uterus weight (in OVEX rats); and the estrogen-responsive liver proteins HDL cholesterol and SHBG (in postmenopausal women).
- In both the experimental animals and the postmenopausal women, the changes in the levels of all estrogenic markers were consistent with the presence of biologically active phytoestrogens in the congeners.
- Red wine congeners and bourbon congeners produced similar estrogenic effects in experimental animals and in postmenopausal women.

Various aspects of the estrogenic effects of alcoholic beverage congeners, however, remain to be elucidated. For example, it is currently unknown whether and how phytoestrogens in alcoholic beverage congeners affect the lining of the uterus and the mineral content of the bones, both of which are influenced by estrogens produced in the body. These issues should be explored in clinical trials in which the participants are randomly assigned to receiving congeners. Furthermore, researchers must address the question of how the phytoestrogenic congeners interact with the alcohol in alcoholic beverages. So far, the effects of these substances have only been investigated separately; however, every person who consumes alcoholic beverages ingests both the alcohol and the congeners. Scientists have yet to determine how these potential interactions can best be evaluated.

## Footnotes

1 A standard drink is defined as a 12-ounce bottle of beer or wine cooler, a 5-ounce glass of wine, or 1.5 ounces of 80-proof distilled spirits.

2 Prolactin is essential for the development and growth of the mammary gland and for the initiation and maintenance of milk production in nursing women. Prolactin production is increased in the presence of estrogen.

## References

[b1-arh-22-3-220] (1998). Bordeaux Symposium. Alcoholism: Clinical and Experimental Research.

[b2-arh-22-3-220] Gavaler JS, Van Thiel DH (1988). Gonadal dysfunction and inadequate sexual performance in cirrhotic alcoholic men. (Editorial.). Gastroenterology.

[b3-arh-22-3-220] Gavaler JS, Imhoff AF, Pohl CR, Rosenblum ER, Van Thiel DH (1987a). Alcoholic beverages: A source of estrogenic substances?. Alcohol and Alcoholism.

[b4-arh-22-3-220] Gavaler JS, Rosenblum ER, Van Thiel DH, Eagon PK, Pohl CR, Campbell IM (1987b). Biologically active phytoestrogens are present in bourbon. Alcoholism: Clinical and Experimental Research.

[b5-arh-22-3-220] Gavaler JS, Rosenblum ER, Deal SR, Bowie BT (1995a). The phytoestrogen congeners of alcoholic beverages: Current status. Proceedings of the Society of Experimental Biology and Medicine.

[b6-arh-22-3-220] Gavaler JS, Rosenblum ER, Deal SR, Watson RR (1995b). Hidden hormones in alcoholic beverages. Drug and Alcohol Abuse Reviews: Volume 6. Alcohol and Hormones.

[b7-arh-22-3-220] Hertog MGL, Hollman PCH, van de Putte B (1993). Content of potentially anticarcinogenic flavonoids of tea infusions, wines and fruit juices. Journal of Agriculture and Food Chemistry.

[b8-arh-22-3-220] Klatsky AL (1994). Epidemiology of coronary heart disease: Influence of alcohol. Alcoholism: Clinical and Experimental Research.

[b9-arh-22-3-220] Makela S, Poutanen M, Lehtimaki J, Kostian ML, Santti R, Vihko R (1995). Estrogen-specific 17B-hydroxysteroid oxidoreductase Type 1 (E>C>1.1.1.62) as a possible target for the action of phytoestrogens. Proceedings of the Society of Experimental Biology and Medicine.

[b10-arh-22-3-220] Miksicek RJ (1995). Estrogenic flavonoids: Structural requirements for biological activity. Proceedings of the Society of Experimental Biology and Medicine.

[b11-arh-22-3-220] Rosenblum ER, Van Thiel DH, Campbell IM, Gavaler JS (1987). Isolation and identification of phytoestrogenic compounds isolated from bourbon. Alcohol and Alcoholism.

[b12-arh-22-3-220] Rosenblum ER, Van Thiel DH, Campbell IM, Gavaler JS (1991). Quantitation of B-sitosterol in bourbon. Alcoholism: Clinical and Experimental Research.

[b13-arh-22-3-220] Rosenblum ER, Stauber RE, Van Thiel DH, Campbell IM, Gavaler JS (1993). Assessment of the estrogenic activity of phytoestrogens isolated from bourbon and beer. Alcoholism: Clinical and Experimental Research.

[b14-arh-22-3-220] U.S. Department of Agriculture and U.S. Department of Health and Human Services (1995). Nutrition and Your Health: Dietary Guidelines for Americans.

[b15-arh-22-3-220] Wright HI, Gavaler JS, Tabasco-Minguillan J, Van Thiel DH, Mendelson J, Mello N (1992). Endocrine effects of alcohol abuse in males. Medical Diagnosis and Treatment of Alcoholism.

